# Relationship between facet tropism and facet joint degeneration in the sub-axial cervical spine

**DOI:** 10.1186/s12891-017-1448-x

**Published:** 2017-02-20

**Authors:** Xin Rong, Ziyang Liu, Beiyu Wang, Xuelin Pan, Hao Liu

**Affiliations:** 1 Department of Orthopedic Surgery, West China Hospital, Sichuan University, No. 37, Guo Xue Xiang, Chengdu, Sichuan Province 610041 China; 2 Department of Radiology, West China Hospital, Sichuan University, Chengdu, Sichuan Province 610041 China

**Keywords:** Tropism, Degeneration, Cervical spine, Facet joint

## Abstract

**Background:**

Facet tropism is the angular asymmetry between the left and right facet joint orientation. Although debatable, facet tropism was suggested to be associated with disc degeneration, facet degeneration and degenerative spondylolisthesis in the lumbar spine. The purpose of this study was to explore the relationship between facet tropism and facet degeneration in the sub-axial cervical spine.

**Methods:**

A total of 200 patients with cervical spondylosis were retrospectively analyzed. Facet degeneration was categorized into 4 grade: grade I, normal; grade II, degenerative changes including joint space narrowing, cyst formation, small osteophytes (<3 mm) without joint hypertrophy; grade III, joint hypertrophy secondary to large osteophytes (>3 mm) without fusion of the joint; grade IV, bony fusion of the facet joints. Facet orientations and facet tropisms with respect to the transverse, sagittal and coronal plane were calculated from the reconstructed cervical spine, which was based on the axial CT scan images. The paired facet joints were then categorized into three types: symmetric, moderated tropism and severe tropism. Univariate and multivariate analysis were performed to evaluate the relationship between any demographic and anatomical factor and facet degeneration.

**Results:**

The mean age of enrolled patients was 46.23 years old (ranging from 30 to 64 years old). There were 114 males and 86 females. The degrees of facet degeneration varied according to cervical levels and ages. Degenerated facet joints were most common at C2-C3 level and more common in patients above 50 years old. The facet orientations were also different from level to level. By univariate analysis, genders, ages, cervical levels, facet orientations and facet tropisms were all significantly different between the normal facets and degenerated facets. However, results from multivariate logistic regression suggested only age and facet tropism with respect to the sagittal plane were related to facet degeneration.

**Conclusion:**

Facet degeneration were more common at C2-C3 level. Older age and facet tropism with respect to the sagittal plane were associated with the facet degeneration.

**Electronic supplementary material:**

The online version of this article (doi:10.1186/s12891-017-1448-x) contains supplementary material, which is available to authorized users.

## Background

Facet tropism is defined as the angular asymmetry between the left and right facet joint orientation [1]. It was postulated that abnormal stress distribution as well as abnormal motion would occur with the presence of facet tropism [2]. Numerous clinical studies suggested that facet tropism could be the predisposing factor for some pathological changes in the lumbar spine, including disc degeneration [3–6], facet degeneration [7, 8] and degenerative spondylolisthesis [9]. However, the relationship between facet tropism and lumbar degenerative changes is still debatable [10–12]. This may due to the heterogeneity among the studies with regard to different patient population and different criteria for the definition of facet tropism.

Interestingly though, there has been no study evaluating the relationship between facet tropism and cervical facet degeneration, as far as we know. The facet joints are synovial joints in the sub-axial cervical spine, which are of great importance guiding the spinal motion and transmitting the axial loading [13, 14]. Besides, the cervical facets are also held responsible for the neck pain to some extent [15, 16]. As with other joints, the cervical facet joints degenerate with age, including cartilage thinning, osteophyte formation or hypertrophy, sclerosis and joint space narrowing [17–19].

The purpose of the present study was to evaluate the relationship between facet tropism and facet degeneration in the sub-axial cervical spine.

## Methods

This was a retrospective study approved by the Ethical Committee of West China Hospital of Sichuan University. Patients were selected from a larger group of 1325 patients, who were diagnosed with spondylotic radiculopathy, spondylotic myelopathy or both at our institution from July 2013 to June 2015. Exclusion criteria was as follow: osteoporosis (T-score lower than −2.5 with or without fracture); tumor or infection at any cervical level; deformity; no available CT data; insufficient CT data (not fully covering C2 to C7 vertebra); slice thickness or slice increment larger than 1 mm. All patients had given the informed consent to allow their information to be used in research purposes.

Facet degeneration was assessed according to a recently published criteria [20]. For each patient, the facet degeneration on both the left and right side from C2-C3 to C6-C7 level were categorized into 4 grades according to articular space, cyst formation, and articular process hypertrophy. Briefly: grade I, normal; grade II, degenerative changes including joint space narrowing, cyst formation, small osteophytes (<3 mm) without joint hypertrophy seen on axial or sagittal images; grade III, joint hypertrophy secondary to large osteophytes (>3 mm) without fusion of the joint seen on sagittal images; grade IV, bony fusion of the facet joints. The CT scans were read by one radiologist and one senior spine resident. We first tested the reliability of the grading system in 20 patients. CT scans were assigned to the two readers in a random sequence at the interval of 2 weeks. The intra-observer and inter-observer reliability was assessed by intraclass correlation (ICC) value (excellent for the ICC value from 0.9 to 1, good for 0.7 to 0.89, fair for 0.5 to 0.69, low for 0.25 to 0.49, poor for 0 to 0.24). In the later part of this study, when two different grading results were presented for one facet joint, the lower grade was assigned as the final grading results, as indicated by the previous study [20]. The facet joints were further categorized into normal (grade I) and degenerated (grade II or above) for later analysis.

**Fig. 1:**
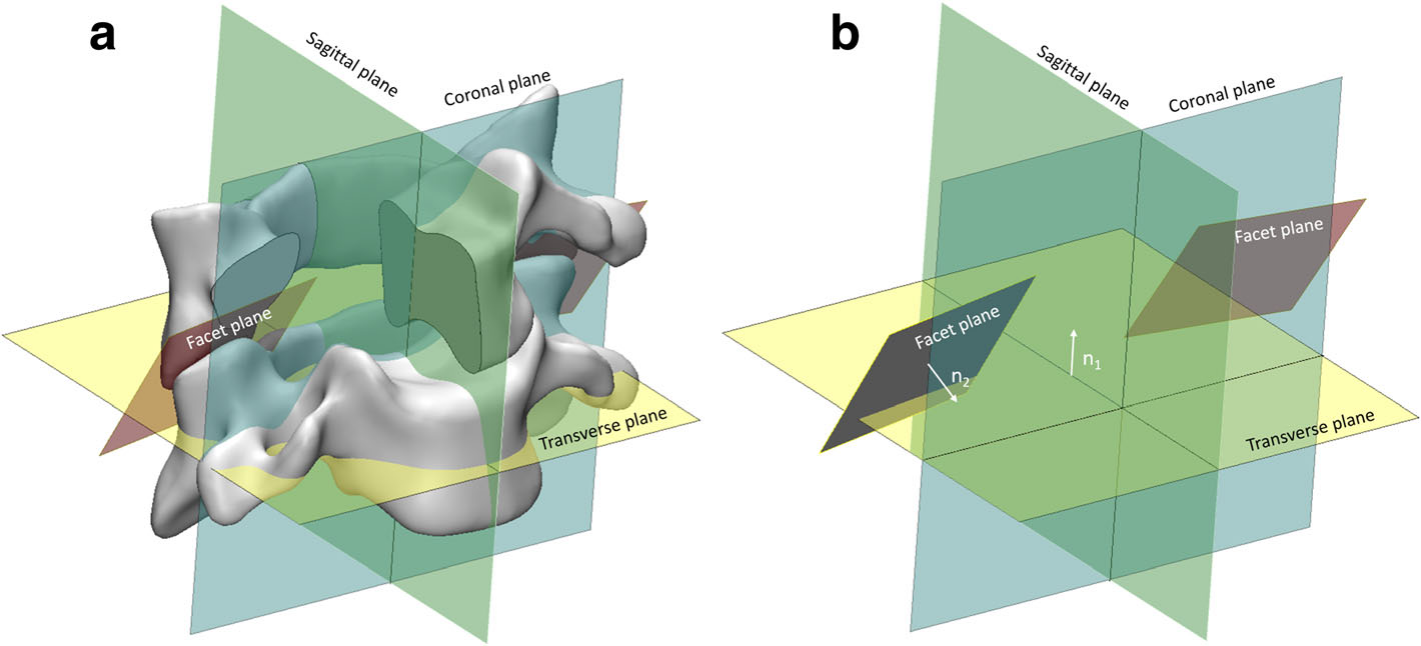
Illustration of the determination of the facet orientations in reconstructed cervical spine. The facet plane bisects the facet joint space; the transverse plane parallel to the superior endplate of the vertebral body and perpendicular to the sagittal plane; the sagittal plane bisects the vertebral body; the coronal plane are perpendicular to both the transverse plane and the sagittal plane (**a**). The normal vectors of one facet plane (n_1_) and transverse plane (n_2_), of which the coordinates were used for the calculation of the angle between the facet plane and transverse plane (**b**)

The facet orientations with respect to the transverse, sagittal and coronal plane were determined on the reconstructed cervical spine (Fig. 1). First, the CT data in DICOM format was imported into the commercially available software Mimics 17.0 (Materialize, Belgium) to reconstruct the cervical spine. Second, five planes were identified on the reconstructed cervical spine: two facet planes, the plane bisects the facet joint space on either side; transverse plane, the plane parallel to the superior endplate of the vertebral body and perpendicular to the sagittal plane; sagittal plane, the plane bisects the vertebral body; and coronal plane, the plane perpendicular to both the transverse plane and the sagittal plane. Third, the normal vectors of the five planes were used to calculate the angles between two planes for the determination of the facet orientations as follows:

$$ \cos\ \alpha = \cos \left({n}_1,{n}_2\right)=\frac{n_1\cdot {n}_2}{\left|{n}_1\right|\cdot \left|{n}_2\right|} $$

where α means the angle between two planes, n_1_ and n_2_ means the normal vectors of the two planes. The inclination of facets with respect to the transverse, sagittal and coronal planes were termed as T-angle, S-angle and C-angle, respectively.

**Fig. 2:**
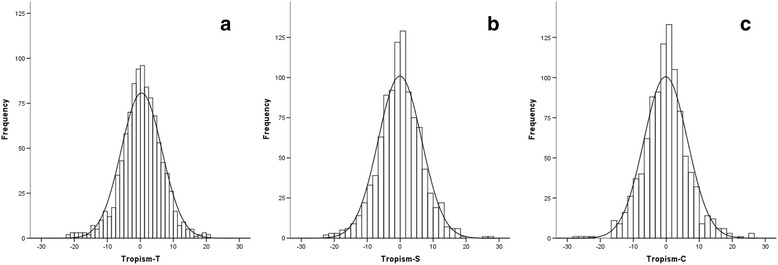
The histogram of the raw difference between the right-side and left-side facet orientations with respect to the transverse plane (tropism-T, with the mean of 0.36 and the SD of 6.18) (**a**); with respect to the sagittal plane (tropism-S, with the mean of −0.07 and the SD of 6.59) (**b**); with respect to the coronal plane (tropism-C, with the mean of −0.18 and the SD of 6.61)(**c**)

The mean and SD of the raw difference between the left-side and right-side T-angle, S-angle and C-angle, were calculated and termed as tropism-T, tropism-S and tropism-C, respectively. The differences were normally distributed around the mean (Fig. 2). Based on the raw differences, the facet tropism was then classified as symmetric (within 1 SD), moderate tropism (between 1 and 2 SD) and severe tropism (beyond 2 SD) as described by Vanharanta et al. [21]. In this study, the mean of tropism-T, tropism-S and tropism-C was 0.36, −0.07, −0.18 with the SD to be 6.18, 6.59, and 6.61. Coincidently the SD for tropism-T, tropism-S and tropism-C were in a close range. Thus, moderate tropism was defined as a raw difference of 7° to 13°, and severe tropism as more than 13°.

### Statistical analysis

Statistical analysis was performed using SPSS (version 19.0, SPSS Inc., Chicago, IL). The facet orientations were presented as mean ± SD. The paired t-tests were used to detect the difference of the facet orientations between the left and right side. The independent t-test were performed to calculate the difference of the facet orientations between male and female. The one-way ANOVA was adopted to assess the difference of the facet orientation from C2-C3 to C6-C7 level. Univariate analysis including independent student t-test and Chi-square test were used to detect the difference of demographic and anatomical factors between normal facets and degenerated facets. The multivariate logistic regression was then performed to estimate the demographic and anatomical factors (age, gender, level, T-angle, S-angle, C-angle, tropism-T, tropism-S and tropism-C) associated with facet degeneration. Further, subgroup analyses according to levels were performed using multivariate logistic regression. A p-value less than 0.05 was deemed statistically significant.

## Results

A total of 200 patients were retrospectively enrolled in this study. The mean age was 46.23 years old (ranging from 30 to 64 years old). There were 114 males with the mean age of 45.95 years old (ranging from 30 to 64 years old) and 86 females with the mean age of 46.60 years old (ranging from 31 to 61 years old). No significant difference was noted between the genders (*P* > 0.05).

The degrees of facet degeneration varied according to levels (Table 1, *P* = 0.000). Degenerated facet joints were most common at C2-C3 level. The degrees of facet degeneration varied according to ages (Table 2, *P* = 0.000). Facet degenerative changes were more common in patients above 50 years old. The intra-observer reliability was 0.867 (95%CI: 0.841 to 0.890) and the inter-observer reliability was 0.757 (95%CI: 0.711 to 0.797), which were of good reliability.

**Table 1:** The grades of facet degeneration according to cervical levels

	C2-C3	C3-C4	C4-C5	C5-C6	C6-C7	Total
Grade I (%)	150 (75)	163 (81.5)	162 (81)	162 (81)	186 (93)	823 (82.3)
Grade II (%)	35 (17.5)	30 (15)	28 (14)	31 (15.5)	14 (7)	138 (13.8)
Grade III (%)	15 (7.5)	7 (3.5)	10 (5)	7 (3.5)	0 (0)	39 (3.9)
Degenerated (%)	50 (25)	37 (18.5)	38 (19)	38 (19)	14 (7)	177 (17.7)
Total (%)	200 (100)	200 (100)	200 (100)	200 (100)	200 (100)	1000 (100)

**Table 2:** The degrees of facet degeneration according to ages

	30–39	40–49	50–59	60–69	Total
Normal (%)	149 (87.65)	432 (88.16)	200 (71.43)	42 (70)	823 (82.3)
Degenerated (%)	21 (12.35)	58 (11.84)	80 (28.57)	18 (30)	177 (17.7)
Total (%)	170 (100)	490 (100)	280 (100)	60 (100)	1000 (100)

The facet orientations on both sides according to cervical levels and genders are summarized in Table 3. No significant difference was noted between the right-side and left-side. Significant differences between genders were noted for right-side T-angle and right-side S-angle at C2-C3 level. Right-side T-angle at C2-C3 level in males was significantly larger than that in females (57.99° ± 7.77° vs 54.73° ± 7.73°, *P* = 0.004). Right-side S-angle at C2-C3 level in males was significantly larger than that in females (73.42° ± 7.73° vs 71.27° ± 6.32°, *P* = 0.032). Significant difference was observed among cervical levels in all facet orientations. Briefly, T-angle was largest at C6-C7 level and S-angle was smallest at C2-C3 level. C-angle was largest at C2-C3 level and smallest at the C6-C7 level.

**Table 3:** Facet orientations according to cervical levels and genders

	T-angle (°)		S-angle (°)		C-angle (°)	
	Right side	Left side	Right side	Left side	Right side	Left side
Male						
C2-C3	57.99 ± 7.77	56.87 ± 8.48	73.42 ± 7.73¶	73.71 ± 8.03¶	39.38 ± 7.77¶	40.23 ± 7.91¶
C3-C4	57.17 ± 5.74	56.44 ± 6.09	84.05 ± 7.98	84.50 ± 8.09	34.79 ± 6.61	35.14 ± 6.67
C4-C5	54.89 ± 6.49	54.7 ± 6.480	90.30 ± 7.88	89.94 ± 8.47	36.23 ± 7.01	36.64 ± 6.81
C5-C6	57.10 ± 6.51	56.69 ± 6.32	91.65 ± 8.78	91.65 ± 7.50	34.40 ± 6.53	34.45 ± 6.08
C6-C7	64.64 ± 6.66¶	63.85 ± 6.16¶	88.94 ± 8.08	89.85 ± 8.89	26.93 ± 6.46¶	27.75 ± 6.55¶
Female						
C2-C3	54.73 ± 7.73*	54.72 ± 9.24	71.27 ± 6.32*¶	72.20 ± 6.76¶	41.19 ± 7.62¶	40.43 ± 9.16¶
C3-C4	56.21 ± 5.74	55.34 ± 6.60	84.28 ± 7.46	84.56 ± 7.20	35.61 ± 6.08	36.01 ± 6.49
C4-C5	55.00 ± 6.81	55.48 ± 5.87	92.17 ± 8.86	91.51 ± 7.84	36.50 ± 6.96	35.68 ± 5.86
C5-C6	55.95 ± 6.60	56.75 ± 5.74	93.45 ± 7.67¶	92.87 ± 8.33¶	35.27 ± 6.62	34.68 ± 6.09
C6-C7	63.39 ± 6.48¶	63.05 ± 7.31¶	90.19 ± 7.30	89.38 ± 7.61	27.76 ± 6.42¶	28.30 ± 7.37¶

*T-angle* the inclination of facets with respect to the transverse plane, *S-angle* the inclination of facets with respect to the sagittal plane, *C-angle* the inclination of facets with respect to coronal plane

* *P* < 0.05 compared to the males

·¶ *P* < 0.05 compared to other levels

No significant difference was noted between right side and left side

Facet tropism with respect to transverse plane and coronal plane varied according to levels (Table 4, *P* = 0.005 for tropism-T and *P* = 0.000 for tropism-C). Tropism-T and tropism-C were most common at C2-C3 level. Facet tropism with respect to sagittal plane was not significantly different among levels (Table 4, *P* = 0.196).

**Table 4:** Severity of facet tropisms according to levels

	C2-C3	C3-C4	C4-C5	C5-C6	C6-C7	P
Tropism-T						0.005
Symmetry (%)	137 (68.5)	165 (82.5)	163 (81.5)	159 (79.5)	157 (78.5)	
Moderate (%)	43 (21.5)	31 (15.5)	31 (15.5)	32 (16)	33 (16.5)	
Severe (%)	20 (10)	4 (2)	6 (3)	9 (4.5)	10 (5)	
Tropism-S						0.196
Symmetry (%)	148 (74)	157 (78.5)	141 (70.5)	155 (77.5)	146 (73)	
Moderate (%)	42 (21)	27 (13.5)	46 (23)	38 (19)	44 (22)	
Severe (%)	10 (5)	16 (8)	13 (6.5)	7 (3.5)	10 (5)	
Tropism-C						0.000
Symmetry (%)	122 (61)	155 (77.5)	157 (78.5)	162 (81)	151 (75.5)	
Moderate (%)	51 (25.5)	35 (17.5)	37 (18.5)	30 (15)	42 (21)	
Severe (%)	27 (13.5)	10 (5)	6 (3)	8 (4)	7 (3.5)	

*Tropism-T* difference between right-side and left-side T-angle, *Tropism-S* difference between right-side and left-side S-angle, *Tropism-C* difference between right-side and left-side C-angle

Univariate comparison of demographic and anatomical factors between normal facets and degenerated facets are summarized in Table 5. All included demographic and anatomical factors between normal facets and degenerated facets were significantly different (*P* < 0.05). Overall association of demographic and anatomical factors is demonstrated in Table 6. Results from multivariate logistic regression suggested that age, gender, cervical levels, tropism-T and tropism-S were associated with facet degeneration. Association of demographic and anatomical factors at individual level with facet degeneration are listed in Table 7. Gender was not associated with facet degeneration except for C2-C3 level, whereas age was related to facet degeneration at all sub-axial levels except for C6-C7 level. Tropism-S were related to facet degeneration at all levels, whereas tropism-T was noted only related to facet degeneration at C2-C3 level.

**Table 5:** Demographic and anatomical factors and univariate analysis for facet degeneration

	Sub-axial cervical facet joints		
	Normal	Degenerated	P
Gender			0.015
Male	484	86	
Female	339	91	
Age			0.000
< 50	581	79	
≥ 50	242	98	
Level			0.000
C2-C3	150	50	
C3-C4	163	37	
C4-C5	162	38	
C5-C6	162	38	
C6-C7	186	14	
T-angle	58.09 ± 6.60	55.40 ± 7.43	0.000
S-angle	86.26 ± 10.04	84.60 ± 10.38	0.047
C-angle	34.27 ± 7.10	37.44 ± 7.56	0.000
Tropism-T			0.000
Symmetric	664	117	
Moderate	132	38	
Severe	27	22	
Tropism-S			0.000
Symmetric	635	112	
Moderate	159	38	
Severe	29	27	
Tropism-C			0.000
Symmetric	636	111	
Moderate	152	43	
Severe	35	23

**Table 6:** Multivariate analysis of factors associated with facet degeneration

	P	OR	95%CI	
Gender				
Male	0.009	1.611	1.126	2.304
Female	Reference			
Age				
< 50	0.000	3.873	2.673	5.610
≥ 50	Reference			
Level				
C2-C3	0.000	0.196	0.081	0.473
C3-C4	0.002	0.312	0.148	0.655
C4-C5	0.004	0.338	0.162	0.703
C5-C6	0.002	0.313	0.153	0.643
C6-C7	Reference			
T-angle	0.750	1.013	0.935	1.098
S-angle	0.328	0.986	0.959	1.014
C-angle	0.782	0.989	0.912	1.072
Tropism-T				
Symmetric	0.001	4.703	1.879	11.769
Moderate	0.009	3.126	1.325	7.376
Severe	Reference			
Tropism-S				
Symmetric	0.000	8.405	4.284	16.490
Moderate	0.000	5.976	2.895	12.334
Severe	Reference			
Tropism-C				
Symmetric	0.901	0.945	0.389	2.300
Moderate	0.869	1.070	0.480	2.383
Severe	Reference			

*OR* odds ratio, *CI* confidence interval

**Table 7:** Multivariate analysis of factors associated with facet degeneration according to levels

		P	OR	95%CI	
C2-C3					
Gender	Male	0.043	2.961	1.037	8.453
	Female	Reference			
Age	<50	0.001	3.410	1.605	7.246
	≥50	Reference			
Tropism-T	Symmetric	0.003	10.998	2.224	54.378
	Moderate	0.003	11.256	2.273	55.739
	Severe	Reference			
Tropism-S	Symmetric	0.022	9.121	1.371	60.661
	Moderate	0.022	9.885	1.399	69.854
	Severe	Reference			
C3-C4					
Age	<50	0.000	5.522	2.273	13.410
	≥50	Reference			
Tropism-S	Symmetric	0.001	10.765	2.697	42.960
	Moderate	0.021	6.774	1.331	34.470
	Severe	Reference			
C4-C5					
Age	<50	0.002	3.944	1.648	9.442
	≥50	Reference			
Tropism-S	Symmetric	0.006	8.352	1.838	37.950
	Moderate	0.358	2.036	0.447	9.284
	Severe	Reference			
C5-C6					
Age	<50	0.000	5.458	2.317	12.857
	≥50	Reference			
Tropism-S	Symmetric	0.005	22.343	2.546	196.109
	Moderate	0.010	21.616	2.118	220.648
	Severe	Reference			
C6-C7					
Tropism-S	Symmetric	0.001	44.059	4.569	424.904
	Moderate	0.001	269.824	8.699	8368.862
	Severe	Reference			

Only those factors with a *p* value smaller than 0.05 is listed here

## Discussion

To our best knowledge, there were two CT-based grading system for facet degeneration [20, 22]. The scoring system proposed by Walraevens et al. based on 20 patients had good intra-observer reliability (ICC = 0.71) and fair inter-observer reliability (ICC = 0.49) [22]. The grading system proposed by Park et al. based on 320 patients had better reliability with the intra-observer agreement of 0.881 and the inter-observer agreement of 0.869 [20]. In this study, we adopted the grading system proposed by Park because of the higher reliability, and re-tested the reliability in a small sample (20 patients). Our investigation reproduced good intra-observer (ICC = 0.867) and inter-observer (ICC = 0.757) reliabilities.

The distribution of facet degeneration among the cervical levels in our study were similar to the previous study [20]. However, the incidence of facet degeneration was higher in our study. The total incidence of facet degeneration above grade II was 17.7% in the present study, whereas the incidence was 8.6% in the study by Park et al. [20]. This may due to the fact that all patients in our study underwent cervical surgeries, suggesting more severe cervical degeneration. However, interestingly, we did not find any grade IV degeneration. We believe this was because the patients in the present study was younger (46.23 years, ranged from 30 to 64 years). In the study by Park et al. [20], the study population was older (60 years, ranged from 40 to 81 years). Besides, the reported incidence of grade IV degeneration was very low (<1%) [20, 23].

Results from the multivariate analysis suggested that several demographic and anatomical factors, including gender, age, cervical level and facet tropism, were associated with facet degeneration in the sub-axial cervical spine.

Several studies suggested that gender was associated with facet degeneration in the cervical spine [20, 23]. Park et al. [20] reported that both facet degeneration above grade II and above grade III were more common in males. Morishita et al. [24] found that hypertrophic change of facet joint occurred more frequently in males. Uhrenholt et al. [19] performed histological observation on 40 subjects and demonstrated that facet cartilage flaking and splitting were more common in males, On the contrary, in the present study, the results from the multivariate analysis when taken the sub-axial cervical spine as a whole showed, that facet degeneration were more common in females. However, in the sub-group analysis according to levels, we found that gender was only related to facet degeneration at C2-C3 level. Therefore, we suggested that gender may not be independently associated with facet degeneration. Nevertheless, cross-sectional study of large sample was needed to verify this finding.

Older age was suggested to be related to facet degeneration. Cadaveric studies demonstrated that the prevalence of cervical facet degenerative changes increased with age, including cartilage thinning, osteophyte formation or hypertrophy, sclerosis and joint space narrowing [17–19]. Park et al. [20] enrolled 320 patients (40 males and 40 females from each of the following age groups: 40 to 49, 50 to 59, 60 to 69, and 70 to 79). They reported that about 3% of the facet joints in patients younger than 60 were degenerated. However, 9.13% of the facet joints in patients older than 60 and 19.13% of the facet joints in patients older than 70 were degenerated. Our study found that about 10% of the facet joints in patients younger than 50 were degenerated, whereas around 30% of the facet joints in patients older than 50 were degenerated. Although the patient population were different in these two studies, same trend was found that facet degenerations were more common in older patients. Results from multivariate analysis according to levels in our study further confirmed that facet degeneration were age related.

Our results demonstrated that facet degeneration were more likely to happen at C2-C3 level. At the other end of the cervical spine, C6-C7 had the lowest incidence of facet degeneration. Park et al. [20] found that C2-C3 to C4-C5 levels had higher incidence of facet degeneration. Morishita et al. [24] reported higher incidence of facet joints hypertrophy at mid-level (C4-C5) of the cervical spine. These results suggested that the facet degenerative changes were more likely to happen in the upper sub-axial cervical levels. It was quite different from the lumbar spine, in which facet degeneration tended to occur at the lower lumbar level [25]. Future studies are needed to elaborate the mechanism behind this phenomenon.

There was one theory that facet tropism could create asymmetrical stress distribution in the facet joints. Biomechanical study by Cyron and Hutton [2] demonstrated that facet tropism caused higher compressive load on the facet joints in axial rotation. Kim et al. [26, 27] concluded in their finite element study that facet tropism could increase the local facet contact force. Such an imbalanced loading could result in the development of facet degeneration, such as osteophytes formation and joint space narrowing. Some clinical studies in the lumbar spine confirmed this theory that facet tropism could be associated with facet degeneration [7]. Shin et al. [7] conducted a retrospective study on 42 patients with 51 lumbar levels replaced with artificial discs. At the 36 months follow-up, the progressive facet arthrosis (PFA) levels had significantly larger facet tropism than the non-PFA levels. However, little is known about the relationship between facet tropism and facet degeneration in the cervical spine. Results from our multivariate analysis suggested that the facet tropism with respect to the sagittal plane seemed to be associated with facet degeneration. Clinical observations and finite element studies are warranted to assess the impact of facet tropism on the cervical facet joints.

Some limitations existed in the present study. Firstly, the findings of this study were based on surgical patients with cervical radiculopathy, myelopathy, or both, which suggested that any association between facet tropism and facet degeneration seen in this population might be different from the asymptomatic population. Secondly, the facet joints and the corresponding intervertebral disc formed the “joint complex”, which meant any pathological changes occurred in the intervertebral disc could affect the facet joints, and vice versa. In this study, the disc degeneration was not taken into consideration. However, there was evidence demonstrating that the presence of Modic changes and facet joint degeneration at the same level of the cervical spine were not related [23]. Nevertheless, it is important for the future studies to take into consideration of the effect of the cervical intervertebral discs. Thirdly, we could not establish the relationship between our findings on CT and occurrence of neck pain. Although patients in our study were all diagnosed with cervical spondylosis, not all of them had neck pain. Besides, neck pain were multifactorial. Pain from facet degeneration require confirmatory facet block. Therefore, abnormal findings on CT scans could not simply imply clinical symptoms.

## Conclusions

Facet tropism was common in the sub-axial cervical spine. Incidence of facet degeneration was highest at C2-C3 level, whereas lowest at C6-C7 level. Facet degeneration was associated with older age and more severe facet tropism with respect to the sagittal plane.

## Additional file

Additional file 1: The original data of the gender, age, cervicl level, grading of the facet degeneration and facet orientations. (XLSX 448 kb)

## Abbreviations

C-angle: The inclination of facets with respect to coronal plane
S-angle: The inclination of facets with respect to the sagittal plane
T-angle: The inclination of facets with respect to the transverse plane
Tropism-C: Difference between right-side and left-side C-angle
Tropism-S: Difference between right-side and left-side S-angle
Tropism-T: Difference between right-side and left-side T-angle

## References

[1] Brailsford JF (1929). Deformities of the lumbosacral region of the spine. Br J Surg.

[2] Cyron BM, Hutton WC (1980). Articular tropism and stability of the lumbar spine. Spine.

[3] Chadha M, Sharma G, Arora SS, Kochar V (2013). Association of facet tropism with lumbar disc herniation. Eur Spine J.

[4] Do DH, Taghavi CE, Fong W, Kong MH, Morishita Y, Wang JC (2011). The relationship between degree of facet tropism and amount of dynamic disc bulge in lumbar spine of patients symptomatic for low back pain. Eur Spine J.

[5] Park JB, Chang H, Kim KW, Park SJ (2001). Facet tropism: a comparison between far lateral and posterolateral lumbar disc herniations. Spine.

[6] Farfan HF, Sullivan JD (1967). The relation of facet orientation to intervertebral disc failure. Can J Surg.

[7] Shin MH, Ryu KS, Hur JW, Kim JS, Park CK (2013). Association of facet tropism and progressive facet arthrosis after lumbar total disc replacement using ProDisc-L. Eur Spine J.

[8] Masharawi Y, Rothschild B, Dar G, Peleg S, Robinson D, Been E, Hershkovitz I (2004). Facet orientation in the thoracolumbar spine: three-dimensional anatomic and biomechanical analysis. Spine.

[9] Dai LY (2001). Orientation and tropism of lumbar facet joints in degenerative spondylolisthesis. Int Orthop.

[10] Murtagh FR, Paulsen RD, Rechtine GR (1991). The role and incidence of facet tropism in lumbar spine degenerative disc disease. J Spinal Disord.

[11] Boden SD, Riew KD, Yamaguchi K, Branch TP, Schellinger D, Wiesel SW (1996). Orientation of the lumbar facet joints: association with degenerative disc disease. J Bone Joint Surg Am.

[12] Linov L, Klindukhov A, Li L, Kalichman L (2013). Lumbar facet joint orientation and osteoarthritis: a cross-sectional study. J Back Musculoskelet Rehabil.

[13] Yoganandan N, Kumaresan S, Pintar FA (2001). Biomechanics of the cervical spine Part 2. Cervical spine soft tissue responses and biomechanical modeling. Clin Biochem.

[14] Yang KH, King AI (1984). Mechanism of facet load transmission as a hypothesis for low-back pain. Spine.

[15] Manchikanti L, Singh V, Rivera J, Pampati V (2002). Prevalence of cervical facet joint pain in chronic neck pain. Pain Physician.

[16] Manchikanti L, Boswell MV, Singh V, Pampati V, Damron KS, Beyer CD (2004). Prevalence of facet joint pain in chronic spinal pain of cervical, thoracic, and lumbar regions. BMC Musculoskelet Disord.

[17] Gallucci M, Puglielli E, Splendiani A, Pistoia F, Spacca G (2005). Degenerative disorders of the spine. Eur Radiol.

[18] Kettler A, Werner K, Wilke HJJ (2007). Morphological changes of cervical facet joints in elderly individuals. Eur Spine J.

[19] Uhrenholt L, Hauge E, Charles AV, Gregersen M (2008). Degenerative and traumatic changes in the lower cervical spine facet joints. Scand J Rheumatol.

[20] Park MS, Lee YB, Moon SH, Lee HM, Kim TH, Oh JB, Riew KD (2014). Facet joint degeneration of the cervical spine: a computed tomographic analysis of 320 patients. Spine.

[21] Vanharanta H, Floyd T, Ohnmeiss DD, Hochschuler SH, Guyer RD (1993). The relationship of facet tropism to degenerative disc disease. Spine.

[22] Walraevens J, Liu B, Meersschaert J, Demaerel P, Delye H, Depreitere B, Vander Sloten J, Goffin J (2009). Qualitative and quantitative assessment of degeneration of cervical intervertebral discs and facet joints. Eur Spine J.

[23] Park MS, Moon SH, Kim TH, Lee SY, Jo YG, Riew KD (2015). Relationship between modic changes and facet joint degeneration in the cervical spine. Eur Spine J.

[24] Morishita K, Kasai Y, Uchida A (2008). Hypertrophic change of facet joint in the cervical spine. Med Sci Monit.

[25] Eubanks J, Lee MJ, Cassinelli E, Ahn NU (2007). Prevalence of lumbar facet arthrosis and its relationship to age, sex, and race: an anatomic study of cadaveric specimens. Spine.

[26] Kim HJ, Chun HJ, Lee HM, Kang KT, Lee CK, Chang BS, Yeom JS (2013). The biomechanical influence of the facet joint orientation and the facet tropism in the lumbar spine. Spine J.

[27] Kim HJ, Kang KT, Son J, Lee CK, Chang BS, Yeom JS (2015). The influence of facet joint orientation and tropism on the stress at the adjacent segment after lumbar fusion surgery: a biomechanical analysis. Spine J.

